# VvWRKY5 enhances white rot resistance in grape by promoting the jasmonic acid pathway

**DOI:** 10.1093/hr/uhad172

**Published:** 2023-08-29

**Authors:** Zhen Zhang, Changyue Jiang, Cui Chen, Kai Su, Hong Lin, Yuhui Zhao, Yinshan Guo

**Affiliations:** College of Horticulture, Shenyang Agricultural University, 120 Dongling Road, Shenyang, Liaoning 110866, China; College of Horticulture, Shenyang Agricultural University, 120 Dongling Road, Shenyang, Liaoning 110866, China; College of Horticulture, Shenyang Agricultural University, 120 Dongling Road, Shenyang, Liaoning 110866, China; College of Horticulture Science and Technology, Hebei Normal University of Science and Technology, Qinhuangdao 066004, China; College of Horticulture, Shenyang Agricultural University, 120 Dongling Road, Shenyang, Liaoning 110866, China; College of Horticulture, Shenyang Agricultural University, 120 Dongling Road, Shenyang, Liaoning 110866, China; College of Horticulture, Shenyang Agricultural University, 120 Dongling Road, Shenyang, Liaoning 110866, China; National & Local Joint Engineering Research Center of Northern Horticultural Facilities Design and Application Technology (Liaoning), Shenyang 110866, China

## Abstract

Grape white rot is a disease caused by *Coniella diplodiella* (Speg.) Sacc. (*Cd*) can drastically reduce the production and quality of grape (*Vitis vinifera*). WRKY transcription factors play a vital role in the regulation of plant resistance to pathogens, but their functions in grape white rot need to be further explored. Here, we found that the expression of the WRKY IIe subfamily member *VvWRKY5* was highly induced by *Cd* infection and jasmonic acid (JA) treatment. Transient injection and stable overexpression (in grape calli and *Arabidopsis*) demonstrated that VvWRKY5 positively regulated grape resistance to white rot. We also determined that VvWRKY5 regulated the JA response by directly binding to the promoters of *VvJAZ2* (a JA signaling suppressor) and *VvMYC2* (a JA signaling activator), thereby inhibiting and activating the transcription of *VvJAZ2* and *VvMYC2*, respectively. Furthermore, the interaction between VvJAZ2 and VvWRKY5 enhanced the suppression and promotion of *VvJAZ2* and *VvMYC2* activities by VvWRKY5, respectively. When *VvWRKY5* was overexpressed in grape, JA content was also increased. Overall, our results suggested that VvWRKY5 played a key role in regulating JA biosynthesis and signal transduction as well as enhancing white rot resistance in grape. Our results also provide theoretical guidance for the development of elite grape cultivars with enhanced pathogen resistance.

## Introduction

Grapes (*Vitis vinifera*) are a popular fruit worldwide; however, they are extremely vulnerable to many diseases, which can result in significant crop losses in viticulture. Grape white rot caused by *Coniella diplodiella* (Speg.) Sacc. (*Cd*) is one of the most serious fungal diseases affecting grape [1]. White rot can infect leaves, berries, and new shoots, among other tissues. The most common entry sites for white-rot pathogens are wounds caused by weather-related incidents, insects, or other fungal illnesses [1]. In many areas afflicted by this disease, grape yields have decreased by at least 16.3% [2]. Fungicides are widely used in viticulture; however, they can have negative effects on food safety, cause environmental pollution, and increase production costs [3]. Therefore, characterizing the genes responsible for grape defense against white rot can offer useful information for breeding resistant varieties.

Plant defense responses are regulated by various signaling pathways, including oxidative bursts [4], MAPK cascades [5], transcription factors (TFs) [6], and changes in phytohormones [7, 8]. Phytohormones salicylic acid (SA) and jasmonic acid (JA) are both key mediators of plant pathogen resistance [9–11]. They can trigger the synthesis of defense-related molecules such as phenolic compounds (like phytoalexins) and pathogenesis-related (PR) proteins [12, 13]. In *Arabidopsis*, SA mainly promotes defensive resistance against biotrophic pathogens such as *Pseudomonas syringae*, whereas JA typically promotes defensive resistance against necrotrophic pathogens such as *Fusarium oxysporum* [14].

When plant cells have low concentrations of JA, a group of JAZ (JASMONATEZIM DOMAIN) proteins can combine with the corepressor TOPLESS to form inhibitory complexes that prevent MYC2, a basic helix–loop–helix (bHLH) protein, from triggering the expression of JA-responsive genes [15]. Additionally, when plants are stimulated by stress, JA accumulates in plant cells, relieving JAZ protein inhibition of MYC2 and activating the expression of JA-responsive genes [16].

WRKY TFs are essential for plant resistance to various diseases. For instance, WRKY transcription factors regulate resistance to diseases, such as witches’ broom disease in Chinese jujube, canker disease in citrus, *Glomerella* leaf spot in apple, downy mildew in grape, and *Ralstonia solanacearum* infection in tobacco [17–21]. To date, 70 WRKY proteins have been identified in *Arabidopsis* [22], as well as more than 100 in rice [23]. WRKY domains and zinc-finger motifs are the two most noticeable structural features of WRKY proteins [24]. Based on the number of WRKY domains and the pattern of the zinc-finger motif, WRKY proteins can be categorized into three primary groups (I–III). Furthermore, Group II is then classified into five subgroups (IIa–IIe) [25]. In addition to the WRKY domains and zinc finger motifs shared by these TFs, some WRKY members have proline-rich regions, glutamine-rich regions, leucine-zipper domains, kinase domains, or nuclear localization signals [26]. This distinctive structural arrangement allows the WRKY TFs to perform various regulatory functions.

In *Arabidopsis*, the WRKY IIa member WRKY18 enhances developmentally regulated defense responses [27]. Group III members WRKY53, WRKY38, and WRKY62 [28–30] have all been found to display more severe sensitivity to *Pst* (*Pseudomonas syringae* pv. tomato) DC3000; however, WRKY41 showed increased resistance [31]. Overexpression of the group III member AtWRKY70 increases resistance to *necrotrophic* infections by inhibiting JA signaling and activating SA signaling [32, 33]. Functional studies have previously revealed that the WRKY III members OsWRKY13 [34], OsWRKY53 [35], OsWRKY89 [36], OsWRKY31 [37], and OsWRKY45 [38] play positive regulatory roles in rice blast resistance. Interestingly, overexpression of the group IIa member *OsWRKY62* reduced resistance to *Xanthomonas oryzae* pv. *oryzae* [39]. In grapevine, many WRKY TFs have been shown to be closely associated with plant disease resistance. For example, overexpression of *VvWRKY1* (WRKY IIc) increases the resistance of transgenic lines to gray mold and downy mildew [40]. *VqWRKY31* (WRKY IIb) enhances grape resistance to powdery mildew by promoting SA signaling and related metabolite synthesis [41]. *VqWRKY53* (WRKY IIc) can promote stilbene biosynthesis and increase resistance to *Pst* DC3000 [42]. Knockout of *VvWRKY52* (WRKY III) can enhance the resistance of transgenic grape to gray mold [43]. Overexpression of *VqWRKY56* (WRKY IIb) in grapevine increased PA content and reduced susceptibility to powdery mildew [44]. Although the functions of several WRKY IIa–IIc and WRKY III members in plant defense have been previously studied, the roles of other subgroup members remain unclear.

Herein, we demonstrate that the WRKY IIe subfamily member VvWRKY5 positively regulates grape white rot resistance by regulating the JA pathway. VvWRKY5 represses the expression of *VvJAZ2* but activates the expression of *VvMYC2* by directly binding to their promoters. Furthermore, the interaction between VvJAZ2 and VvWRKY5 enhances the suppression and promotion of *VvJAZ2* and *VvMYC2* activities by VvWRKY5, respectively. When *VvWRKY5* was overexpressed in grape, the content of JA was also increased. Our findings provide a strong theoretical background for future research aimed at developing elite grape cultivars with enhanced pathogen resistance.

**Figure 1:**
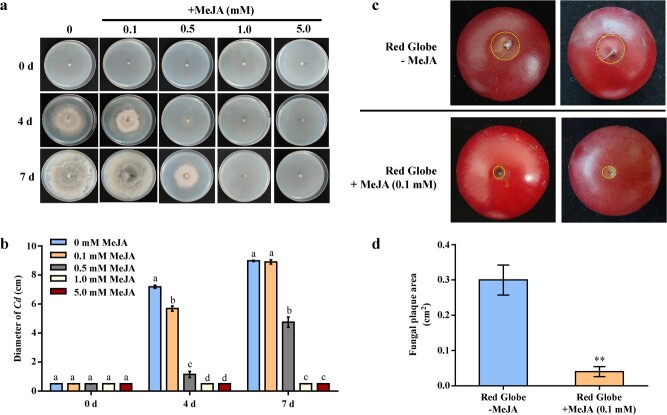
Effect of MeJA treatment on *Cd* resistance in grapes. **a** MeJA affects *Cd* growth. *Cd* mycelial disks (1 cm diameter) were cultured on PDA medium supplemented with 0, 0.1, 0.5, 1.0, and 5.0 mM MeJA for 7 days. **b** Statistical analysis of the *Cd* mycelium growth rate shown in (**a**). Values are means ± standard deviation of three replicates. Statistical significance is indicated by different lowercase letters (*P* < .05). **c** Phenotype of ‘Red Globe’ fruit (with or without 0.1 mM MeJA treatment) inoculated with *Cd* for 3 days. **d** Determination of plaque area of ‘Red Globe’ fruit after being inoculated with *Cd*. Values are means ± standard deviation of three replicates. Tukey’s test was used for detecting significant differences using DPS software (^**^P < .01).

**Figure 2:**
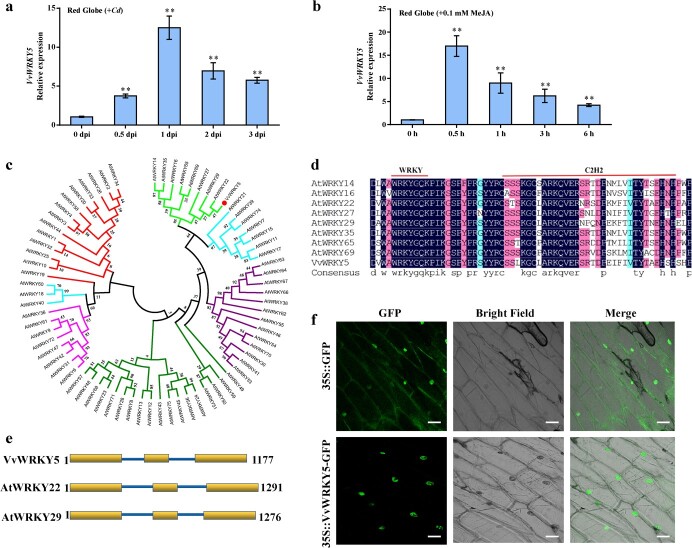
Responses of *VvWRKY5* to *Cd* and MeJA treatments. **a** Expression of *VvWRKY5* in ‘Red Globe’ fruit after being inoculated with *Cd*. The density of *Cd* spore suspension used to inoculate the fruits was 1 × 10^7^/ml. **b** Expression of *VvWRKY5* in ‘Red Globe’ fruit under 0.1 mM MeJA treatment. Values are means ± standard deviation of three replicates. Tukey’s test was used for detecting significant differences using DPS software (^**^P < .01). **c** Phylogenetic analysis of VvWRKY5 and 75 members of the *Arabidopsis* WRKY family. Accession numbers are listed in Supplementary Data Table S4. **d** Multiple sequence alignment of VvWRKY5 with eight IIe subfamily members of *Arabidopsis*. **e** Genomic structures of VvWRKY5, AtWRKY22, and AtWRKY29. Boxes represent exons and lines represent introns. **f** Subcellular localization of VvWRKY5. Onion epidermal cells were transformed with the VvWRKY5-GFP plasmid. Scale bars, 25 μm.

**Figure 3:**
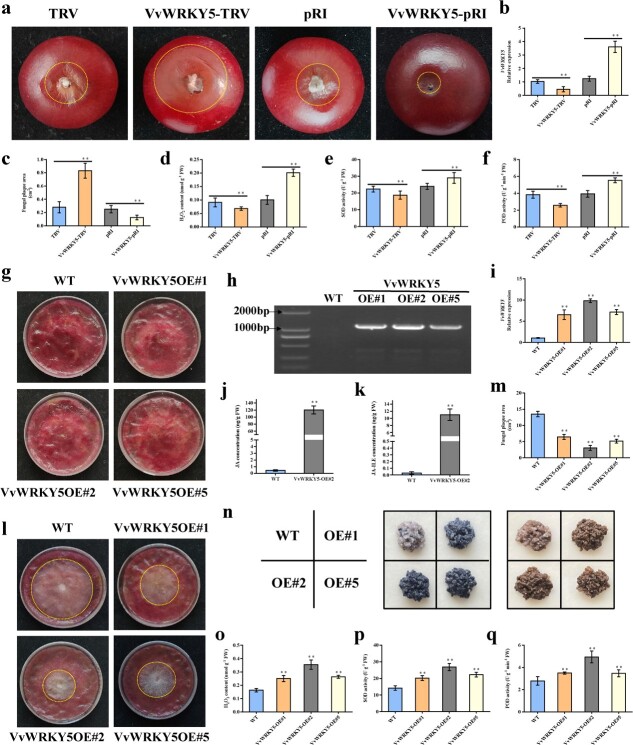
VvWRKY5 enhances grape resistance to white rot. **a** Phenotype of ‘Red Globe’ fruit (*VvWRKY5* transiently silenced or overexpressed) inoculated with *Cd* for 3 days. The density of *Cd* spore suspension used to inoculate ‘Red Globe’ fruits was 1 × 10^7^/ml. **b** Expression of *VvWRKY5* in transiently transformed grape fruits. **c** Determination of plaque area of ‘Red Globe’ fruit after inoculation with *Cd*. **d**–**f** H_2_O_2_ content (**d**), SOD activity (**e**), and POD activity (**f**) of ‘Red Globe” fruit after being inoculated with *Cd*. **g** Phenotype of WT and *VvWRKY5*-overexpressing grape calli (VvWRKY5-OE#1/2/5). **h**, **i** Expression levels of DNA (**h**) and RNA (**i**) to detect stable *VvWRKY5*-overexpressing grape calli. **j**, **k** Quantification of JA (**j**) and JA-ILE (**k**) concentration in the WT and VvWRKY5-OE#2 grape calli. **l** Phenotype of WT and *VvWRKY5-*overexpressing grape calli after being infected with *Cd* for 2 days. The density of *Cd* spore suspension used to inoculate the grape calli was 1 × 10^7^/ml. **m** Determination of plaque area of WT and *VvWRKY5-*overexpressing calli after being inoculated with *Cd*. **n** DAB and NBT staining of WT and *VvWRKY5*-overexpressing calli after being infected with *Cd*. **o**–**q** H_2_O_2_ content (**o**), SOD activity (**p**), and POD activity (**q**) of WT and *VvWRKY5*-overexpressing calli infected with *Cd*. Values are means ± standard deviation of three replicates. Tukey’s test was used for detecting significant differences by DPS software (^**^P < .01).

**Figure 4:**
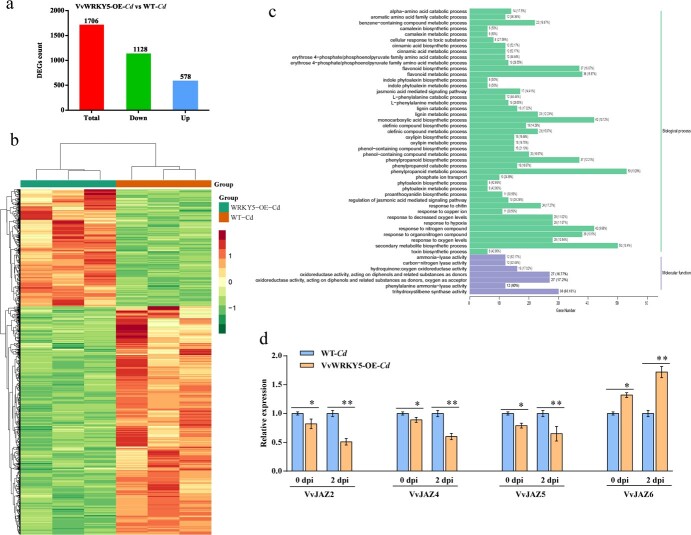
RNA-seq analysis of the role of the VvWRKY5 regulator in *Cd* response. **a** Number of DEGs among VvWRKY5-OE-*Cd* vs WT-*Cd* at 2 dpi by RNA-seq. VvWRKY5-OE-*Cd*, VvWRKY5-OE#2 grape calli after infection with *Cd*; WT-*Cd*, wild-type grape calli after infection with *Cd*. **b** Heat map of DEGs based on RNA-seq data of VvWRKY5-OE-*Cd* vs WT-*Cd* at 2 dpi. **c** GO enrichment analysis of DEGs in VvWRKY5-OE-*Cd* vs WT-*Cd* at 2 dpi. **d** Expression of *VvMYC2*, *VvJAZ2*, *VvJAZ4*, and *VvJAZ6* in WT and VvWRKY5-OE#2 grape calli at 0 and 2 dpi with *Cd*. Values are means ± standard deviation of three replicates. Tukey’s test was used for detecting significant differences using DPS software (^*^P < .05, ^**^P < .01).

**Figure 5:**
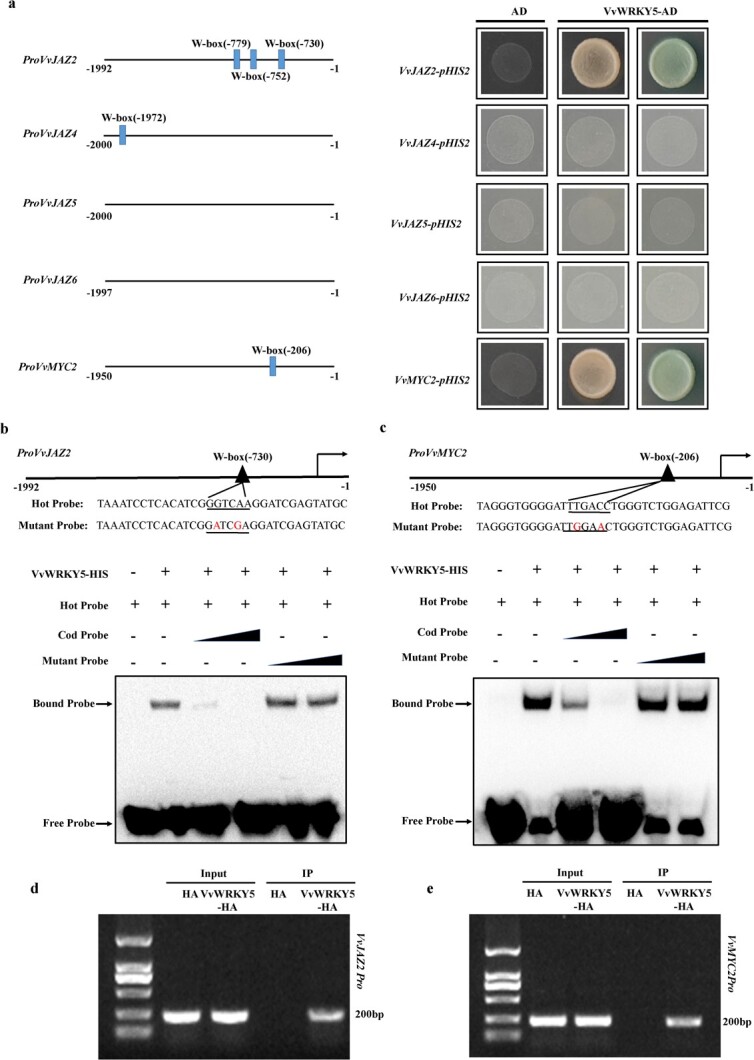
VvWRKY5 binds to the promoters of *VvJAZ2* and *VvMYC2*. **a** Y1H assays. The AD empty vector and the *VvJAZ2*, *VvJAZ4*, *VvJAZ5*, *VvJAZ6*, and *VvMYC2* promoters were used as negative controls. **b**, **c** EMSA assays showed the binding of VvWRKY5 to the *VvJAZ2* (**b**) and *VvMYC2* (**c**) promoters. Hot probes were biotin-conjugated promoter fragments with specific W-box motifs. Cold probe was an unlabeled competitive probe. Mutant probes were unlabeled fragments containing mutated nucleotides of the W-box motifs of *VvJAZ2* and *VvMYC2*. Cold and mutant probes were added in increasing amounts (100× or 200×). **d**, **e** ChIP–PCR assays confirmed the binding of VvWRKY5 to the *VvJAZ2* (**d**) and *VvMYC2* (**e**) promoters. The DNA fragments enriched in each ChIP served as the biological replicate in PCR.

**Figure 6:**
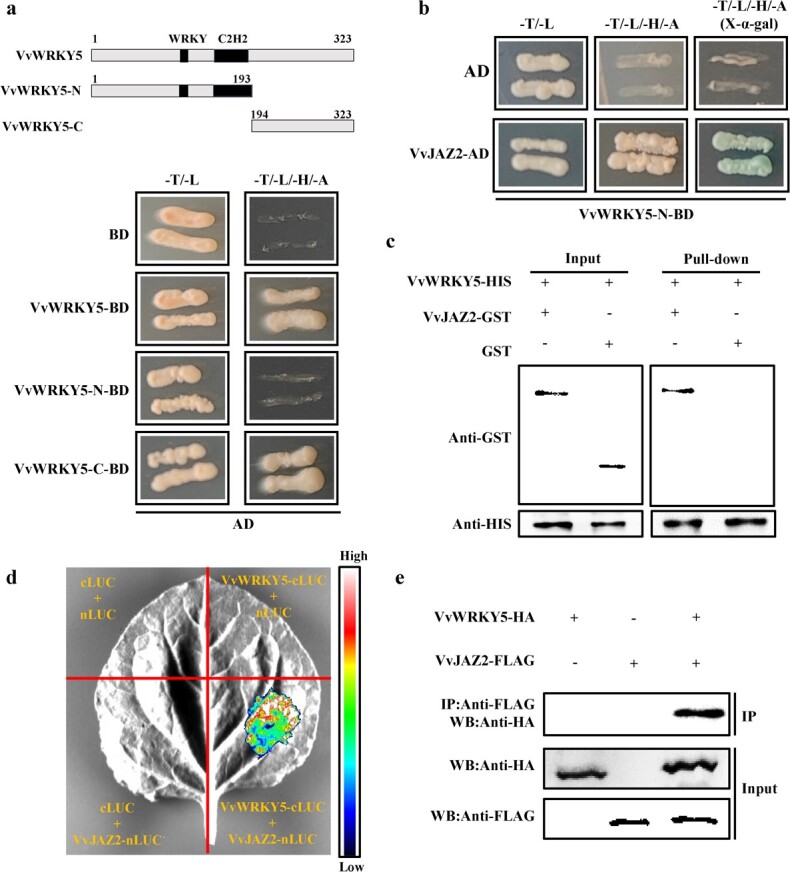
VvWRKY5 interacts with VvJAZ2. **a** Y2H assays showed that the VvWRKY5-C terminal is self-activated. VvWRKY5, full-length VvWRKY5; VvWRKY5-N, N-terminal of VvWRKY5; VvWRKY5-C, C-terminal of VvWRKY5. **b** Y2H assays showed that VvWRKY5 interacted with VvJAZ2. −T/–L, SD medium lacking Trp and Leu; −T/−L/−H/−A, SD medium lacking Trp, Leu, His, and Ade; X-α-gal, SD medium containing X-α-gal. The empty AD vector was used as the negative control. **c** Pull-down assay. The GST antibody-bound band indicates an interaction between VvWRKY5 and VvJAZ2. **d** VvWRKY5 interacted with VvJAZ2 in *in vivo* LCI assays. **e** Co-IP assays showing the interaction between VvWRKY5 and VvJAZ2 *in vivo*. The HA antibody-bound band indicates an interaction between VvWRKY5 and VvJAZ2.

## Results

### Jasmonic acid inhibited *Cd* growth and enhanced *Cd* resistance in grape berries

Exogenous application of MeJA (a stable derivative of JA) and SA can both activate the defense mechanisms of certain plant against pathogenic organisms [45, 46]. We cultured *Cd* mycelia in the same growth period and state on PDA plates containing different concentrations of MeJA and SA. Both MeJA (Fig. 1a and b) and SA (Supplementary Data Fig. S1) treatments were found to have inhibited *Cd* growth. However, compared with SA, MeJA had a stronger bacteriostatic effect at the same concentration (Fig. 1a and b; Supplementary Data Fig. S1). Furthermore, the higher the concentration of MeJA, the stronger its inhibitory effect on *Cd* megaspore formation (Fig. 1a and b). These findings demonstrated that MeJA treatment inhibited the growth of *Cd* more effectively.

Because 0.1 mM of MeJA appears to be a relatively low concentration but effectively inhibited the growth of *Cd* (Fig. 1a and b), we tested the effect of exogenous 0.1 mM MeJA treatment on *Cd* resistance in grape. After treating the ‘Red Globe’ grape fruits with or without 0.1 mM MeJA, we then inoculated them with *Cd*. We subsequently found that exogenous MeJA treatment effectively slowed *Cd* diffusion in grape fruits (Fig. 1c and d). Specifically, at 3 days post infection (dpi), non-MeJA-treated grape fruits began to rot and die from the top down, which was accompanied by the spread of white mycelia, whereas MeJA-treated fruits showed no evident necrosis or decomposition (Fig. 1c). Meanwhile, the fungal plaque area in MeJA-treated grape fruits was found to be ~80% smaller than that of untreated fruits (Fig. 1d). These results indicated that MeJA treatment enhanced *Cd* resistance in grape.

### *VvWRKY5* was highly induced by *Cd* and MeJA

WRKY TFs play a key role in plant disease resistance [47, 48]. To identify the WRKY genes linked to grape white rot, the expression of 59 VvWRKY genes [49] after *Cd* infection in grape fruits was examined. RT–qPCR results showed that *VvWRKY5* was highly induced after *Cd* inoculation (Supplementary Data Fig. S2). We also monitored the expression of *VvWRKY5* in ‘Red Globe’ fruits during *Cd* infection. At 1 dpi, the expression of *VvWRKY5* was induced almost 12-fold (Fig. 2a). Furthermore, we found that after 0.5 h of MeJA treatment *VvWRKY5* was induced ~17-fold in ‘Red Globe’ (Fig. 2b). These results indicated that *VvWRKY*5 was strongly induced by *Cd* and MeJA treatments in grape.

Phylogenetic analysis revealed that VvWRKY5 belongs to clade IIe of the WRKY TF family and was most closely related to AtWRKY22 and AtWRKY29 in *Arabidopsis* (Fig. 2c). AtWRKY22 and AtWRKY29 are required for resistance to fungi and bacteria [50, 51]. Amino acid homology analysis showed that VvWRKY5 contains a WRKY domain and a C2H2 zinc-finger motif (Fig. 2d). Three exons and two introns constitute the genomic sequence of VvWRKY5, similar to the exon–intron structure of AtWRKY22 and AtWRKY29 (Fig. 2e). Transient expression of the VvWRKY5-GFP protein in onion epidermal cells revealed that VvWRKY5 was localized within the nucleus (Fig. 2f), indicating the potential role of VvWRKY5 in transcriptional regulation.

### VvWRKY5 played a positive role in resistance to *Cd*

To explore whether VvWRKY5 contributed to *Cd* resistance in grape, we conducted transient transfection for silencing and overexpressing *VvWRKY5* in ‘Red Globe’ fruit mediated by *Agrobacterium* infection (Fig. 3a and b). Compared with the control, transiently silencing *VvWRKY5* significantly reduced *Cd* resistance in grape, whereas, in contrast, overexpressing *VvWRKY5* enhanced *Cd* resistance (Fig. 3a and c). Plants can limit, or even kill, pathogens by inducing ROS production [52]. Therefore, we also measured the H_2_O_2_ content, superoxide dismutase (SOD) activity, and peroxidase (POD) activity near the injection site of grape fruits on the third day after *Cd* infection. The results showed that *VvWRKY5*-pRI fruits had higher H_2_O_2_ content, SOD activity, and POD activity than those of the control, whilst the *VvWRKY5*-TRV fruits were lower in these aspects (Fig. 3d–f). These findings indicated that VvWRKY5 improved grape resistance to *Cd*.

To further confirm these results, we obtained three stable *VvWRKY5*-overexpressing ‘Gamay’ grape calli (VvWRKY5-OE#1/2/5) (Fig. 3g–i). We subsequently found that the content of endogenous JA and JA-isoleucine (JA-ILE) in VvWRKY5-OE#2 was higher than that in the wild type (WT) (Fig. 3j and k), indicating that VvWRKY5 may can increase JA content. The severity of *Cd* infection in transgenic grape calli was assessed by spore infection. At 2 dpi, the fungal plaque areas of VvWRKY5-OE grape calli were smaller than those of the WT (Fig. 3l and m), which was consistent with observations in grape fruits (Fig. 3a and c). Staining with 3,3′-diaminobenzidine (DAB) and nitro blue tetrazolium (NBT) showed that VvWRKY5-OE grape calli accumulated more ROS (Fig. 3n). The H_2_O_2_ content, SOD activity, and POD activity were also all higher in VvWRKY5-OE grape calli than in the WT (Fig. 3o–q). Similarly, three *VvWRKY5*-overexpressing *Arabidopsis* lines (VvWRKY5-OE#1/2/8) were obtained (Supplementary Data Fig. S4a and b) and their *Cd* resistance was tested. We found that the overexpression of *VvWRKY5* in *Arabidopsis* considerably boosted *Cd* resistance (Supplementary Data Fig. S4c–f). Furthermore, by identifying the resistance to *Cd* of 11 different grape varieties, we found that the expression level of *VvWRKY5* in resistant varieties was significantly higher than that in susceptible varieties, indicating that the high *Cd* resistance of resistant grapevine varieties may be closely related to the high expression of *VvWRKY5* after infection (Supplementary Data Fig. S5). Overall, these results indicated that VvWRKY5 positively regulated *Cd* resistance in grape and *Arabidopsis*.

### VvWRKY5 regulated the transcription of jasmonic acid pathway-related genes

To further explore how VvWRKY5 regulated *Cd* resistance in grape, RNA-seq was performed on VvWRKY5-OE-*Cd* (VvWRKY5-OE#2 grape calli after infection with *Cd*) and WT-*Cd* (WT grape calli after infection with *Cd*) at 2 dpi. A total of 277 878 400 clean readings were obtained after low-quality reads were removed. The percentages of Q30 and GC were respectively 92.47–93.35 and 44.33–44.99% (Supplementary Data Table S1), demonstrating the high credibility of the transcriptome sequencing data.

We examined the expression of 31 963 genes in VvWRKY5-OE*-Cd* and WT-*Cd* plants at 2 dpi using RNA-seq and obtained 1706 differentially expressed genes (DEGs). Compared with WT-*Cd*, 578 genes were upregulated whilst 1128 genes were downregulated in VvWRKY5-OE-*Cd* (Fig. 4a and b; Supplementary Data Table S2). Using the Gene Ontology (GO) and Kyoto Encyclopedia of Genes and Genomes (KEGG) databases, we functionally annotated and categorized these 1706 genes. The significantly enriched GO terms of DEGs in VvWRKY5-OE-*Cd* vs WT-*Cd* included ‘phenylpropanoid metabolic process’, ‘jasmonic acid mediated signaling pathway’, ‘monocarboxylic acid biosynthetic process’, and ‘regulation of jasmonic acid mediated signaling pathway’ (Fig. 4c). KEGG enrichment analysis also showed that these DEGs were enriched in ‘metabolic pathways’, ‘biosynthesis of secondary metabolites’, ‘plant hormone signal transduction’, ‘MAPK signaling pathway-plant’, and ‘plant–pathogen interaction’ (Supplementary Data Fig. S6). These findings overall suggested that VvWRKY5 regulated complex biological pathways in grape following *Cd* infection. Moreover, RT–qPCR showed that after *Cd* infection the expression levels of VvJAZ6 were upregulated, whereas *VvJAZ2*, *VvJAZ4*, and *VvJAZ5* were all downregulated in VvWRKY5-OE#2 calli compared with the WT (Fig. 4d), which was consistent with the RNA-seq results.

### VvWRKY5 participated in jasmonic acid-mediated disease resistance by binding to the *VvJAZ2* and *VvMYC2* promoters

The expression levels of VvJAZs were considerably altered in the VvWRKY5 transgenic calli after *Cd* infection (Fig. 4d). To investigate whether VvWRKY5 enhanced *Cd* resistance by directly binding to these genes, we selected *VvJAZ2*, *VvJAZ4*, *VvJAZ5*, and *VvJAZ6*, which were significantly inhibited or induced, as candidate genes. Considering that MYC2 is an important part of the JA signaling pathway [53–55], we also selected *VvMYC2* as a candidate gene.

Interactions between VvWRKY5 and their promoters were examined using yeast one-hybrid (Y1H) assays. As shown in Fig. 5a, VvWRKY5 could bind to the promoters of *VvJAZ2* and *VvMYC2*, but not *VvJAZ4*–*6*. WRKY TFs recognize the W-box (TTGACC) in the promoters of their target genes [56]. The promoters of *VvJAZ2* (Supplementary Data Fig. S7), *VvJAZ4* (Supplementary Data Fig. S8), and *VvMYC2* (Supplementary Data Fig. S11) contained three, one, and one W-box elements, respectively. However, no W-box elements were detected in the *VvJAZ5* (Supplementary Data Fig. S9) and *VvJAZ6* (Supplementary Data Fig. S10) promoters. To determine the *VvJAZ2* promoter region interacting with VvWRKY5, we divided the *VvJAZ2* promoter sequence into two segments (*VvJAZ2pro1*, −748 to −1 bp; *VvJAZ2pro2*, −1992 to −749 bp), with these fragments being used for Y1H experiments. As shown in Supplementary Data Fig. S12, the region that interacted with VvWRKY5 was *VvJAZ2pro1*, which contained one W-box element. Next, we designed electrophoretic mobility shift assay (EMSA) probes using W-box elements. The EMSAs showed that VvWRKY5 could bind to the W-box motifs of the *VvJAZ2* (Fig. 5b) and *VvMYC2* (Fig. 5c) promoters, respectively. To determine whether VvWRKY5 binds to the *VvJAZ2* and *VvMYC2* promoters *in vivo*, chromatin immunoprecipitation PCR (ChIP–PCR) analysis was conducted. The promoter fragments of *VvJAZ2* (Fig. 5d) and *VvMYC2* (Fig. 5e) were significantly enriched in VvWRKY5-OE grape calli. These results indicated that VvWRKY5 bound to the *VvJAZ2* and *VvMYC2* promoters *in vitro* and *in vivo*.

### Physical interaction between VvWRKY5 and VvJAZ2

Previous research has demonstrated that WRKY40 can interact with JAZ proteins (JAZ1–8) to form protein complexes, thereby increasing the resistance to *Phytophthora sojae* through JA signaling [57]. To further reveal the molecular mechanism by which VvWRKY5 regulates the JA signaling pathway, yeast two-hybrid (Y2H) assays were conducted here to test the interactions between VvWRKY5 and VvJAZ proteins (VvJAZ1–6). Strong self-activation was observed in both the VvWRKY5 full-length sequence and C-terminal sequence (1–201 aa), but not in the N-terminal sequence (202–331 aa) (Fig. 6a). Y2H assays also showed that yeast cells co-transformed with VvWRKY5-N-BD and VvJAZ2-AD could grow in SD/−T/−L/−H/−A medium (Fig. 6b), indicating an interaction between VvWRKY5 and VvJAZ2. In contrast, VvWRKY5 did not interact with VvJAZ1, VvJAZ3, VvJAZ4, VvJAZ5, or VvJAZ6 (Supplementary Data Fig. S13). Furthermore, pull-down assays showed that VvJAZ2-GST was pulled down by VvWRKY5-HIS (Fig. 6c), indicating an interaction between VvWRKY5 and VvJAZ2 *in vitro*. Subsequent luciferase complementation imaging (LCI) experiments showed that the fluorescence signal generated by the co-expression of VvWRKY5-cLUC and VvJAZ2-nLUC in tobacco leaves was stronger than that generated in the control (Fig. 6d). Additionally, co-immunoprecipitation (Co-IP) assays using VvWRKY5-HA- and VvJAZ2-FLAG-transfected ‘Garmay’ grape calli were performed *in vivo*. The results of this showed that VvWRKY5-HA coprecipitated with VvJAZ2-FLAG (Fig. 6e). Overall, these observations indicated that VvWRKY5 interacted with VvJAZ2 both *in vitro* and *in vivo*.

### Synergistic effects of VvWRKY5 and VvJAZ2 on *VvJAZ2* and *VvMYC2* expression

**Figure 7:**
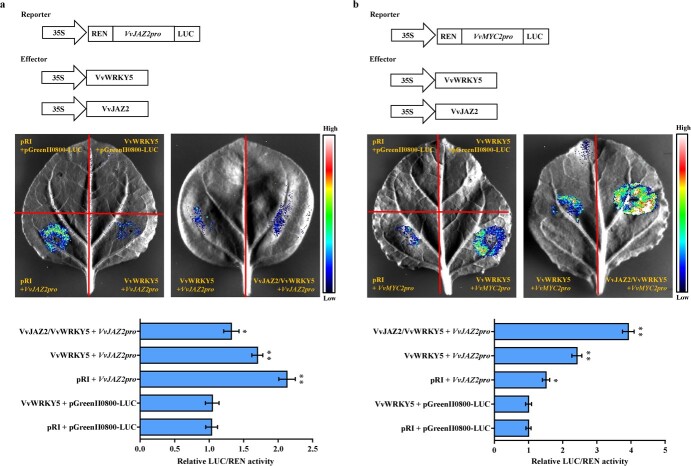
VvJAZ2 enhances the transcriptional regulatory activity of VvWRKY5 on its target genes *VvJAZ2* and *VvMYC2*. **a**, **b** Dual-luciferase reporter assays showed that VvJAZ2 enhances the inhibition and activation effects of VvWRKY5 on *VvJAZ2* (**a**) and *VvMYC2* (**b**) activities, respectively. The LUC/REN ratio of pRI + pGreenII0800-LUC was set to 1. Values are means ± standard deviation of three replicates. Tukey’s test was used for detecting significant differences using DPS software (^*^P < .05, ^**^P < .01).

Based on the observed interactions between VvWRKY5 and VvJAZ2, we hypothesized that VvJAZ2 affects the transcriptional activity of VvWRKY5. We conducted transient dual-luciferase experiments in *Nicotiana benthamiana* using a luciferase (LUC) reporter gene driven by *VvJAZ2* or *VvMYC2* promoters. The LUC results revealed that VvWRKY5 directly inhibited and activated the *VvJAZ2* and *VvMYC2* promoters, respectively. When VvJAZ2 was coexpressed with VvWRKY5, the inhibitory effect of VvWRKY5 on the *VvJAZ2* promoter and the activation effect of VvWRKY5 on the *VvMYC2* promoter were significantly enhanced (Fig. 7a and b), indicating that VvJAZ2 enhanced the transcriptional regulatory activity of VvWRKY5.

**Figure 8:**
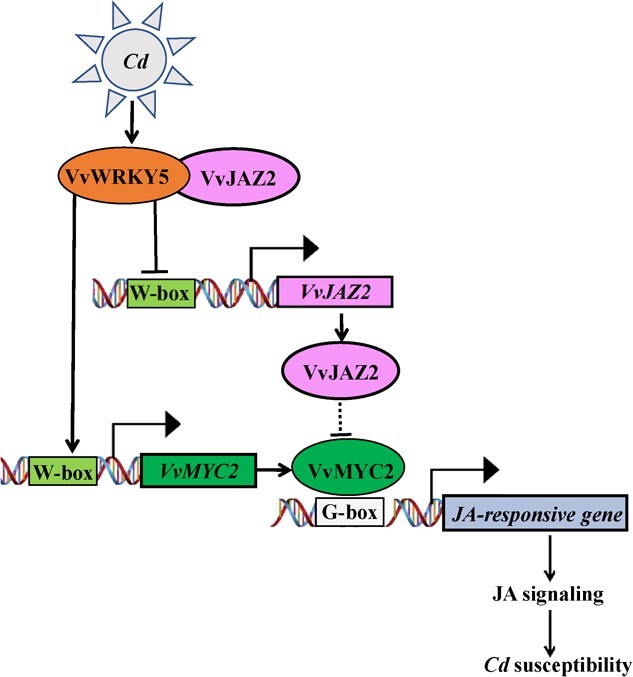
Proposed model showing the role of VvWRKY5 in JA-mediated white rot resistance in grape. VvWRKY5 binds to the promoters of *VvJAZ2* and *VvMYC2*, repressing *VvJAZ2* transcription and activating *VvMYC2* transcription, thereby enhancing the resistance of grape to *Cd*. In addition, VvJAZ2 interacts with VvWRKY5 and enhances the transcriptional activities of VvWRKY5 to *VvJAZ2* and *VvMYC2*. →, promotion; ⊥, suppression.

## Discussion

The JAZ-MYC module is a core signaling module involved in the transmission of JA signaling [58]. Herein, we demonstrated that VvWRKY5, which belongs to the WRKY IIe subfamily, positively regulated white rot resistance in grape by regulating the JAZ-MYC module. VvWRKY5 not only directly inhibited the expression of *VvJAZ2* but also directly activated the expression of *VvMYC2* (Figs 5 and 7). Additionally, VvWRKY5 physically interacted with VvJAZ2 to form a protein complex, thereby enhancing the inhibition of VvJAZ2 activity and the activation of VvMYC2 activity (Figs 6 and 7). Collectively, our results showed that VvWRKY5 positively regulated grape white rot resistance by mediating the JA signaling pathway (Fig. 8).

The same regulatory genes can positively or negatively regulate different target genes. In apple, MdMYB306-like activates and inhibits the promoter activities of the target genes *MdMYB17* and *MdDFR*, respectively [59]. MdERF2 activates *MdACS1* promoter activity [60] but represses *MdACS6* promoter activity [61]. In this study, VvWRKY5 suppressed and activated the promoter activities of *VvJAZ2* and *VvMYC2*, respectively. However, the mechanisms through which MdMYB306-like, MdERF2, and VvWRKY5 exert opposing regulatory effects on different target genes remain unclear. We hypothesized that their ability to bind specifically to different target gene promoters serves as the primary mechanism by which they perform various regulatory roles. To further investigate the similarities and differences in the regulatory effects, it is necessary to obtain structural information on the binding of regulatory genes to target genes.

Plants are frequently attacked by pathogens; however, only a few can colonize specific hosts, indicating the existence of strong recognition and defense systems in plants. To distinguish between these pathogens and respond appropriately, plants use phytohormones for signal transduction [62]. We found that the expression level of *VvWRKY5* was significantly upregulated after exogenous JA treatment (Fig. 2b), indicating that VvWRKY5 responded to JA signaling. In addition, JA and JA-ILE accumulated in *VvWRKY5*-overexpressing grape calli (Fig. 3j and k). Although we identified the molecular mechanisms of VvWRKY5-mediated JA signal transduction, the mechanism by which VvWRKY5 regulated JA biosynthesis in grape remains worth studying in the future.

Notably, unlike the conditions prior to infection, the contents of JA and JA-ILE in VvWRKY5-OE#2 transgenic grape calli after *Cd* infection were lower than those in the WT calli (Supplementary Data Fig. S3a and b). Previous studies have reported that JA and SA pathways have antagonistic effects on plant defense responses [63]. Considering this phenomenon, we examined the SA content in WT and VvWRKY5-OE#2 grape calli after *Cd* infection. As hypothesized, the SA content in the VvWRKY5-OE#2 grape calli was found to be higher than that in the WT calli after *Cd* infection (Supplementary Data Fig. S3c). Although JA-mediated defense responses can help plants adapt to various biotic and abiotic stresses, they may also have adverse effects on plant growth if these reactions continue to be excessive and are not terminated in time. Based on these results, we believe that this might be a mechanism of homeostatic regulation in plants. Moreover, our results confirmed that VvWRKY5 plays an essential role in the synergistic crosstalk between JA and SA. Many WRKY TFs have previously been reported to play significant regulatory functions in both SA and JA signaling pathways. For example, CaWRKY27 positively regulates tobacco resistance to *R. solanacearum* by regulating SA-, JA-, and ethephon-mediated pathways [17].

In addition to positively regulating resistance to *Cd*, we also found that VvWRKY5 had a positive defense function against the hemibiotrophic pathogen *Pst* DC3000 (Supplementary Data Fig. S14), indicating that VvWRKY5 may be a broad-spectrum resistance gene. Crosstalk between hormonal pathways offers enormous regulatory potential for plants and may also allow them to adjust their defense responses to different invaders [64]. VvWRKY5 plays an important role in the synergistic crosstalk between JA and SA, which may result in a synergistic role for VvWRKY5 in plant defense against necrotrophic and hemibiotrophic pathogens.

ROS networks also play an important role in signaling plant disease resistance [52]. When plants are infected with a pathogen, ROS are quickly produced, thereby preventing the pathogen from entering cells or inducing resistant genes that inhibit pathogen growth [65]. DAB and NBT staining also showed that *VvWRKY5*-overexpressing grape calli and *Arabidopsis* accumulated more ROS than the controls after *Cd* infection (Fig. 3n; Supplementary Data Fig. S4d). Moreover, GO enrichment analysis demonstrated that, after *Cd* infection, the DEGs in VvWRKY5-OE-*Cd* vs WT-*Cd* were related to ‘oxidoreductase activity’ (Fig. 4c), indicating that VvWRKY5 may have promoted antioxidant enzyme activity by regulating these DEGs. Furthermore, KEGG analysis revealed that some DEGs were related to ‘cysteine and methionine metabolism’ (Supplementary Data Fig. S6). Thiols of cysteine and methionine residues are vulnerable to nucleophilic attack by ROS. These data overall indicated that VvWRKY5 could also affect the expression of genes associated with the ROS-mediated defense pathway in grape. Interestingly, we found that the SOD and POD activities were also higher in *VvWRKY5*-overexpressing grape calli than the WT after Cd infection (Fig. 3p and q). Although SOD and POD are generally considered to be ROS-scavenging enzymes, different types of pathogens, different pathogenesis, different sampling time, and other complex factors may cause the relationship between SOD/POD and ROS to be more complex.

Plant defense responses are complicated processes involving a variety of physiological, pathological, and molecular mechanisms, with transcriptional regulation being an important part of plant pathogen defense. In this study, we identified the role of VvWRKY5 in regulating the JA pathway to improve the grape defense response against *Cd*. Since our knowledge of the interaction between JA signaling and *Cd* primarily comes from studies in *Arabidopsis* and grape, and different *Cd* accessions may adopt different infection mechanisms for invading plants, our results provide theoretical guidance for the development of elite grape cultivars with improved pathogen resistance.

## Materials and methods

### Plant materials and growth conditions

The grape cultivars ‘Red Globe’ and ‘Gamay’ grape calli were used in this study. ‘Red Globe’ fruits were harvested from the grape experimental garden at Shenyang Agricultural University, Shenyang, Liaoning, China (41°50′ N, 123°24′ E), and ‘Gamay’ grape calli were cultured as previously described by Rachel *et al*. [66]. ‘Gamay’ grape calli were cultured in the light on a B5 medium (0.2 mg/l kinetin, 2% sucrose, 250 mg/l casein hydrolysate, 100 mg/l myoinositol, 0.1 mg/l NAA, pH 5.8) at 24°C.

*Arabidopsis* (Col-0) and *N. benthamiana* plants were grown in an incubator with 16 h light/8 h dark periods at 25°C.

### RT–qPCR and RNA-seq

Total RNA was isolated using the RNA Plant Plus Reagent Kit (Tiangen, Beijing, China), and cDNA was synthesized using a PrimeScript™ RT Reagent Kit (TaKaRa, Dalian, China). Furthermore, a 7500 Real-Time PCR System (Applied Biosystems) was used for RT–qPCR. *VvActin* (GenBank number XM_002278316.4) was used as the internal control, and the expression levels were calculated using the 2^-ΔΔCt^ method [67]. Supplementary Data Table S3 shows the details of all primers used.

WT and VvWRKY5-OE#2 grape calli after infection with *Cd* at 2 dpi were sampled to extract total RNA. The Illumina HiSeq platform (Metware Biotechnology, Wuhan, China) was used for sequencing. The *Vitis* genome sequences (https://urgi.versailles.inra.fr/Species/Vitis/Data-Sequences/Genome-sequences) were used to align the RNA-seq reads.

### Transformation of grape calli with *VvWRKY5*

The coding sequence (CDS) of *VvWRKY5* was inserted into the pRI101-GFP vector. Grape calli were transformed as previously described by Jia *et al*. [68]. In brief, grape calli were incubated with *Agrobacterium tumefaciens* carrying recombinant plasmids for 30 min, then co-cultured in the dark in B5 medium without antibiotic at 24°C for 2 days. The transformed calli with the VvWRKY5-pRI vector were cultured in B5 medium containing 40-mg/l paromomycin sulfate and 300-mg/l cefotaxime sodium in the light at 24°C.

### Transient expression of *VvWRKY5* in grapes

The *VvWRKY5* CDS was inserted into the pRI101 vector for overexpression, whilst a 388-bp fragment of *VvWRKY5* was inserted into the pTRV2 vector for silencing. The recombinant plasmids VvWRKY5-pRI and VvWRKY5-pTRV were then integrated into *A. tumefaciens* GV3101, respectively [69]. Then, each transformed *A. tumefaciens* was injected into ‘Red Globe’ fruits [70]. The injected ‘Red Globe’ fruits were incubated in the dark for 2 days and then used for pathogen infection experiments. The pericarp near the injection site was used for physiological index determination. Ten injected fruits were used as a biological replicate. Each fruit injection assay was performed using three biological replicates.

### Pathogen infection assay

The *Coniella diplodiella* strain WR01 was grown on PDA medium in a 28°C incubator for 7 days before use. ‘Gamay’ grape calli and ‘Red Globe’ fruits were inoculated with a 1 × 10^7^/ml white rot spore suspension. All infected fruits were incubated in an incubator at 28°C with 95% RH. The infected grape calli and fruit samples were incubated for 2 and 5 days, respectively.

### *Arabidopsis* transformation and disease resistance analysis

*Arabidopsis* was transformed with *A. tumefaciens* GV3101 carrying the VvWRKY5-pRI plasmid with *T*_3_ transgenic lines being used for pathogen infection analysis. Four-week-old *Arabidopsis* leaves were injected with a *Pst* DC3000 spore suspension as previously described [71].

### DAB/NBT staining and measurements of hydrogen peroxide content and superoxide dismutase and peroxidase activities

DAB and NBT staining were performed as described previously by Song *et al*. [72]. The H_2_O_2_ content and SOD and POD activities were measured using commercial kits (Ke Ming Biotechnology, Suzhou, China).

### Yeast one-hybrid assay

The *VvWRKY5* CDS was then inserted into the pGADT7 vector (VvWRKY5-AD). The *VvJAZ2*, *VvJAZ5*, and *VvMYC2* promoters were cloned into pHIS2 vectors (VvJAZ2-pHIS2, VvJAZ5-pHIS2, and VvMYC2-pHIS2, respectively). Yeast Y187 cells carrying recombinant plasmids were then spotted onto SD/−Trp/−Leu/−His medium supplemented with 3-AT and X-α-gal to observe their interactions. Supplementary Data Table S3 shows the details of all primers used here.

### Electrophoretic mobility shift assay

The EMSA was performed as described by Zhang *et al*. [73]. The *VvWRKY5* CDS was cloned into the pET-32a (+) vector. The fusion vector was transfected into BL21 (DE3) cells to obtain the VvWRKY5-HIS protein. An Ni-agarose His-Tag Protein Purification Kit (CWbio, Beijing, China) was used to purify the proteins. A LightShift Chemiluminescent EMSA Kit (Beyotime, Shanghai, China) was used to identify the interaction between the VvWRKY5-HIS protein and the *VvJAZ2*/*VvMYC2* promoter. Supplementary Data Table S3 shows the details of all primers and probes used here.

### ChIP–PCR analysis

The *VvWRKY5* CDS was cloned into the pCB302-HA vector. *Agrobacterium tumefaciens* EHA105 carrying VvWRKY5-HA plasmid was used for ‘Gamay’ grape calli transformation. ChIP–PCR analysis was performed using transgenic grape calli and an EZ-ChIP Chromatin Immunoprecipitation Kit (Upstate, MA, USA). PCR was subsequently used to amplify the DNA fragments with primers including specific binding sequences in the *VvJAZ* and *VvMYC2* promoters. Supplementary Data Table S3 shows the details of all primers used here.

### Dual-luciferase reporter assay

The *VvWRKY5* CDS was inserted into the pRI101 vector as an effector. The *VvJAZ2* and *VvMYC2* promoter fragments were inserted into the pGreenII0800-LUC vector as reporters. The helper plasmid pSoup was used to transform the recombinant vectors into the *A. tumefaciens* GV3101 before being injected into *N. benthamiana* leaves. Luciferase signaling was observed using a live fluorescence imager (Tanon-5200, Shanghai, China), whilst LUC/REN activity was also measured using a luciferase detection kit (Beyotime, Shanghai, China). Supplementary Data Table S3 shows the details of all primers used here.

### Yeast two-hybrid assay

The *VvJAZ1–6* CDSs were inserted into the pGADT7 vector (VvJAZ1/2/3/4/5/6-AD). Full-length *VvWRKY5*, *VvWRKY5-C*, and *VvWRKY5-N* were cloned into pGBKT7 vectors (VvWRKY5-BD, VvWRKY5-C-BD, and VvWRKY5-N-BD, respectively). The recombinant vectors were inserted into Y2H Gold yeast cells and cultured on SD/−Trp/−Leu/−His/−Ade medium to observe their interactions. Supplementary Data Table S3 shows the details of all primers used here.

### Pull-down assay

The *VvWRKY5* and *VvJAZ2* CDSs were inserted into the pET-32a (+) and pGEX-4 T-1 vectors, respectively. The fusion vectors were inserted into BL21 (DE3) cells to obtain the VvWRKY5-HIS and VvJAZ2-GST proteins. A commercial kit (CWbio, Beijing, China) was subsequently used to purify these proteins. Furthermore, Western blotting was performed to identify the eluted products using anti-GST and anti-HIS antibodies (TransGen Biotech, Beijing, China). Supplementary Data Table S3 shows the details of all primers used here.

### Luciferase complementation imaging assay

The *VvWRKY5* CDS was inserted into pCAMBIA1300-cLUC, whilst the *VvJAZ2* CDS was inserted into pCAMBIA1300-nLUC [74]. *Nicotiana benthamiana* was infiltrated with *A. tumefaciens* GV3101 carrying the VvWRKY5-cLUC and VvJAZ2-nLUC constructs. Luciferase signaling in the infiltrated *N. benthamiana* leaves was observed 48 h later using a living fluorescence imager (Tanon-5200, Shanghai, China). Supplementary Data Table S3 shows the details of all primers used here.

### Co-immunoprecipitation assay

The *VvWRKY5* CDS was inserted into the pHBT-AvrRpm1-HA vector (VvWRKY5-HA), whereas the *VvJAZ2* CDS was ligated into a pHBT-AvrRpm1-FLAG vector (VvJAZ2-FLAG). These vectors were transiently inserted into protoplasts derived from ‘Gamay’ grape calli and then incubated for 6 h. Anti-FLAG-agarose beads (Abmart, Shanghai, China) were used for protein purification. The IP buffer was used to wash the beads, whilst anti-FLAG and anti-HA antibodies (Abmart, Shanghai, China) were used to detect the proteins by western blotting. Supplementary Data Table S3 shows the details of all primers used here.

### Quantification of jasmonic acid, jasmonic acid-isoleucine, and salicylic acid

Calli of WT and VvWRKY5-OE#2 inoculated with *Cd* were sampled at 2 dpi. JA, JA-ILE, and SA were all extracted and measured by MetWare Biotechnology (https://www.metware.cn/) using the AB Sciex QTRAP 6500 LC–MS/MS platform.

### MeJA and salicylic acid treatment of *Cd*

MeJA and SA were dissolved in 50% ethanol to obtain a 10 mM stock solution. Subsequently, *Cd* mycelial disks (1 cm diameter) were removed from the 8-cm diameter position on each plate and then cultured on new PDA media supplemented with 0, 0.1, 0.5, 1.0, and 5.0 mM MeJA or SA.

### Statistical analysis

Each experiment was performed in triplicate. Values represent the means ± standard deviation of the three replicates. Tukey’s test was used to detect significant differences using DPS software.

## Acknowledgements

We thank Ying Zhang from Zhengzhou Fruit Research Institute for providing the *Coniella diplodiella* strain WR01. We thank Haifeng Jia from Nanjing Agricultural University for providing the ‘Gamay’ grape calli. The research was supported by the National Natural Science Foundation of China (grant no. 31972368), the China Agriculture Research System (grant no. CARS-29-yc-6), the Basic research innovation capability enhancement project of provincial colleges and universities (grant no. 2021JK06), the Department of Science and Technology of Liaoning Province (grant no. 2022030723-JH5/104), and the Shenyang Science and Technology Bureau Funds (grant no. 21-116-3-27).

## Author contributions

Y.G., H.L., and Z.Z. designed the research. Z.Z., C.J., and C.C. performed the experiments. Z.Z., C.J., C.C., K.S., and Y.Z. analyzed the data. Z.Z., H.L., and Y.G. wrote and modified the manuscript.

## Data availability

All relevant data are available within the article and its supplementary data.

## Conflict of interest statement

None declared.

## Supplementary data

Supplementary data is available at *Horticulture Research* online.

## Supplementary Material

Web_Material_uhad172
