# Supplementary material for: VvWRKY5 enhances white rot resistance in grape by promoting the jasmonic acid pathway
Source: Hortic Res. 2023 Aug 29;10(10):uhad172. doi: 10.1093/hr/uhad172 (PMC10569242; doi:10.1093/hr/uhad172)
Supplement: Web_Material_uhad172 [file web_material_uhad172.zip › Supplementary Data Fig. S1-14.doc]

**Supplementary Data Fig. S1–S14**

**Figure S1.** SA affects the growth of *Cd*. **a** SA affects *Cd* growth. *Cd* mycelial discs (1-cm diameter) were cultured on PDA mediums containing 0, 0.1, 0.5, 1.0, and 5.0 mM SA for 7 d. **b** Statistical analysis of the mycelia growth rate shown in (a). Values are means ± SD of 3 replicates. Statistical significance is indicated by different lowercase letters (*P* < 0.05).

**Figure S2.** Expression analyses of 59 WRKY genes in ‘Red Globe’ fruits after infection with *Cd* for 3 d. The WRKY genes with high homology were determined using primers based on previous research [44]. *VvWRKY5* is marked with a red box. *VvActin* was used as a reference gene. Values are means ± SD of 3 replicates. Tukey’s test was used for detecting the significant differences using DPS software (**P* < 0.05, ***P* < 0.01).

**Figure S3.** Detection of hormone contents after *Cd* infection. **a**–**c** Quantification of JA (**a**), JA-ILE (**b**), and SA (**c**) concentrations in the WT and VvWRKY5-OE#2 grape calli after *Cd* infection. Values are means ± SD of 3 replicates. Tukey’s test was used for detecting the significant differences using DPS software (**P* < 0.05, ***P* < 0.01).

**Figure S4.** Overexpression analysis of *VvWRKY5* in Arabidopsis under artificial inoculation with *Cd*. **a**, **b** The expression levels of DNA (**a**) and RNA (**b**) to identify the stable *VvWRKY5*-overexpressed Arabidopsis lines. Col-0. wild-type; VvWRKY5-OE#1/2/8, *VvWRKY5*-overexpressing Arabidopsis lines. **c** Phenotype of Col-0 and *VvWRKY5*-overexpressing Arabidopsis lines after infection with *Cd* for 3 d. **d**–**f** DAB and NBT staining (**d**), H_2_O_2_ content (**e**), and total chlorophyll content (**f**) of the leaves of Col-0 and *VvWRKY5*-overexpressing Arabidopsis lines after infection with *Cd* for 3 d. Values are means ± SD of 3 replicates. Tukey’s test was used for detecting the significant differences using DPS software (**P* < 0.05, ***P* < 0.01).

**Figure S5.** Analysis of *VvWRKY5* expression in different grapevine cultivars. **a** Leaf phenotypes of different grapevine cultivars inoculated with *Cd* for 3 d. **b** Expression analysis of *VvWRKY5* in different grapevine cultivars shown in (a). Values are means ± SD of 3 replicates. Tukey’s test was used for detecting the significant differences using DPS software (**P* < 0.05, ***P* < 0.01).

**Figure S6.** KEGG enrichment analysis based on RNA-Seq. KEGG enrichment analysis of DEGs based on the RNA-seq data of VvWRKY5-OE-*Cd* vs. WT-*Cd* at 2 dpi. The number of genes classified within each term and the p-values of the terms are shown. In total, 1,706 genes were functionally annotated and classified using the KEGG database.

**Figure S7.** Nucleotide sequence of the *VvJAZ2* promoter. Red letters indicate the W-box motifs.

**Figure S8.** Nucleotide sequence of the *VvJAZ4* promoter. Red letters indicate the W-box motif.

**Figure S9.** Nucleotide sequence of the *VvJAZ5* promoter. There is no W-box motif.

**Figure S10.** Nucleotide sequence of the *VvJAZ6* promoter. There is no W-box motif.

**Figure S11.** Nucleotide sequence of the *VvMYC2* promoter. Red letters indicate the W-box motif.

**Figure S12.** VvWRKY5 interacts with *VvJAZ2-pro1* in Y1H assays. Blue colonies indicate the interaction between VvWRKY5 and *VvJAZ2-pro1*.

**Figure S13.** The interactions between VvWRKY5 and VvJAZ family proteins. No interaction between VvWRKY5 and VvJAZ family proteins, except VvJAZ2, evidenced by Y2H assays. The empty pGADT7 vector (AD) was used as a negative control.

**Figure S14.** Overexpression analysis of *VvWRKY5* in Arabidopsis under artificial inoculation with *Pst* DC3000. **a** Phenotype of Col-0 and *VvWRKY5*-overexpressing Arabidopsis lines (VvWRKY5-OE#1/2/8) after infection with *Pst* DC3000 for 3 d. **b**–**d** DAB and NBT staining (**b**), H_2_O_2_ content (**c**), and total chlorophyll content (**d**) of the leaves of Col-0 and *VvWRKY5*-overexpressing Arabidopsis lines after infection with *Pst* DC3000 for 3 d. Values are means ± SD of 3 replicates. Tukey’s test was used for detecting the significant differences using DPS software (**P* < 0.05, ***P* < 0.01).
